# Generation and *in vivo* characterization of a chimeric α_v_β_5_-targeting antibody 14C5 and its derivatives

**DOI:** 10.1186/2191-219X-3-25

**Published:** 2013-04-04

**Authors:** Caroline Dumolyn, Steve Schoonooghe, Lieselotte Moerman, Sara Neyt, Jurgen Haustraete, Filip De Vos

**Affiliations:** 1 Laboratory of Radiopharmacy, University of Ghent, Harelbekestraat 72, Ghent, 9000, Belgium; 2 Laboratory of Myeloid Cell Immunology, VIB, Pleinlaan 2, Brussels, 1050, Belgium; 3 Laboratory of Cellular and Molecular Immunology, Vrije Universiteit Brussel, Pleinlaan 2, Brussels, 1050, Belgium; 4 Protein Service Facility, Department for Molecular Biomedical Research, VIB, University of Ghent, Technologiepark 927, Ghent, 9052, Belgium

**Keywords:** 14C5 antibody, Integrin α_v_β_5_, Oncology, RID, RIT

## Abstract

**Background:**

Previous studies showed that radiolabeled murine monoclonal antibody (mAb) 14C5 and its Fab and F(ab')_2_ fragments, targeting α_v_β_5_ integrin, have promising properties for diagnostic and therapeutic applications in cancer. To diminish the risk of generating a human anti-mouse antibody response in patients, chimeric variants were created. The purpose of this study was to recombinantly produce chimeric antibody (chAb) derivatives of the murine mAb 14C5 and to evaluate the *in vitro* and *in vivo* characteristics.

**Methods:**

*In vitro* stability, specificity, and affinity of radioiodinated chAb and fragments (Iodo-Gen method) were examined on high-expressing α_v_β_5_ A549 lung tumor cells. *In vivo* biodistribution and pharmacokinetic characteristics were studied in A549 lung tumor-bearing Swiss Nu/Nu mice.

**Results:**

Saturation binding experiments revealed high *in vitro* affinity of radioiodinated chAb, F(ab')_2_, and Fab, with dissociation constants (*K*_D_) of 1.19 ± 0.19, 0.68 ± 0.10, and 2.11 ± 0.58 nM, respectively. ChAb 14C5 showed highest tumor uptake (approximately 10%ID/g) at 24 h post injection, corresponding with other high-affinity Abs. ChF(ab')_2_ and chFab fragments showed faster clearance from the blood compared to the intact Ab.

**Conclusions:**

The chimerization of mAb 14C5 and its fragments has no or negligible effect on the properties of the antibody. *In vitro* and *in vivo* properties show that the chAb 14C5 is promising for radioimmunotherapy, due to its high maximum tumor uptake and its long retention in the tumor. The chF(ab')_2_ fragment shows a similar receptor affinity and a faster blood clearance, causing less non-specific retention than the chAb. Due to their fast blood clearance, the fragments show high potential for radioimmunodiagnosis.

## Background

Tumorigenesis and cancer progression are multi-step processes. During cancer progression the ability to invade and metastasize is a very important aggravating factor. Modulation of crosstalk between cells and/or cell extracellular matrix (ECM) adhesive components is of key importance for the occurrence of metastasis [1]. This modulation includes the release of proteases that remodel the ECM, the deposition of new ECM molecules, and alterations in the expression of cell adhesion molecules such as integrins. Integrins are a family of transmembrane heterodimer glycoprotein cell surface receptors primarily responsible for exchanging information between cells and the surface surrounding ECM. Overexpression of certain integrins on tumor cells, tumor stroma, or neovasculature makes these receptors attractive targets for specific cancer therapy. Two interesting targets in this context are the vascular integrins α_v_β_3_ and α_v_β_5_ for which it is still unclear if they promote [2] or inhibit [3] neovascularization. However, antibodies targeting these molecules can inhibit neovasculature formation, making anti-α_v_β_3_ and anti-α_v_β_5_ antibody-based immunotherapy a viable option [4,5]. Abegrin™, for example, is a humanized monoclonal antibody (mAb) specific for α_v_β_3_, which has anti-angiogenic, tumor growth-inhibiting, and anti-bone metastatic capacities [6-10]. The murine mAb 14C5 binds the integrin α_v_β_5_, a close relative of α_v_β_3_. *In vitro* and *in vivo* studies showed the radioiodinated murine mAb 14C5 to have promising properties for diagnostic and therapeutic applications against integrin α_v_β_5_-expressing tumor cells and/or tumor surrounding stromal cells, for example, fibroblasts [11-14].

Full-sized Abs need to overcome some obstacles before penetrating into a tumor. Although big molecules are able to extravasate out of the ‘leaky vessels’ near the tumor, penetration of Abs into the tumor can still be hampered by some physiological barriers, such as high interstitial pressure and a ‘binding site barrier’ [15,16]. In order to improve the penetration within tumor masses, fragments of mAb 14C5 have been created and evaluated. *In vitro* and *in vivo* studies demonstrated the efficient tumor targeting properties of murine ^131^I-Fab and ^131^I-F(ab')_2_ 14C5 fragments [17]. Despite the promising pre-clinical results, the clinical application of Ab 14C5 and its derivatives could be hampered due to its murine origin. The development of a human anti-mouse Ab (HAMA) response can result in tachyphylaxis, i.e., reduced therapeutic effect caused by reduced targeting of the murine Ab due to immune complex formation after repetitive administration of the Ab. More severe, but rare, side effects of a HAMA response can range from hypersensitivity to life-threatening anaphylactic reactions [18,19]. In order to diminish the risk of generating a HAMA response after Ab-based therapy and thus avoid the associated disadvantages, the murine Ab sequence is made to better resemble human Abs. Chimerization is the first major step towards a humanized molecule and includes the substitution of the mouse heavy and light constant regions with human Ab counterparts [20,21].

In the current study, the mAb 14C5 was chimerized to reduce the risk of HAMA responses in patients. Due to alterations in the chimeric (ch) Ab sequences, conformational changes, changes in affinity, and/or alterations of *in vivo* characteristics can occur as compared to the established characteristics of the murine variant. Therefore, the *in vitro* and *in vivo* targeting properties of the chAb and its fragments were investigated to confirm their α_v_β_5_ targeting properties *in vitro* and *in vivo*.

## Methods

### Cells

HEK293T, a human embryonic kidney cell line transfected with the SV40 large T-Ag (SV40T^tsA1609^) [22], was used for transient eukaryotic expression. A549 is a α_v_β_5_^+^ non-small cell lung cancer cell line [13]. Both cell lines were grown in DMEM containing 10% fetal bovine serum (FBS) (Lonza, Verviers, Belgium). Colo16 is a α_v_β_5_^−^ squamous cell carcinoma cell line [13] grown in RPMI 1640 medium supplemented with 10% FBS. All cells were cultured in an incubator at 37°C with 5% CO_2_.

### Expression plasmids

The pES31Hneo and pES33Ezeo expression plasmids have been described previously [23]. Restriction enzymes were purchased from Fermentas (St. Leon-Rot, Germany), primers (see Additional file 1) from Invitrogen (Life Technologies, Merelbeke, Belgium), and DNA-modifying enzymes and Vent-DNA polymerase from Biolabs (Beverly, MA, USA). All vector constructs were sequence-verified after cloning. The 14C5 VH region was amplified from the murine 14C5 heavy chain [24] by polymerase chain reaction (PCR) with primers 14C5 VH B SOE and NM 101F, and the PH1 CH1 was amplified from the IgG1 PH1 human Ab heavy chain [23] with PH1 CH1 F SOE and NM 263 B. After splice overlap extension (SOE) PCR, the product was cut with BspEI and XhoI, and introduced in the pES31Hneo vector. The chimeric kappa light chain was cloned in a pES33 vector in a similar way, using the NM 101F, 14C5 VL B SOE, PH1 CL F SOE, and NM 264 B primers. SOE PCR was followed by a BspEI and XhoI digest and introduced in the vector. The Fd fragment was further extended to a complete Ab heavy chain by SOE PCR extension with primers PH1 CH1 F SOE, CH1 Hi B, CH2 F, CH2 B, CH3 F, and CH3 B, using pBRhIG1 as template for the hinge, CH2, and CH3 regions. After digestion with AgeI and BamHI enzymes, the product was ligated in the pES33 vector. The heavy chain of the chF(ab')_2_ fragment was amplified by PCR based on the heavy-chain plasmid of the chAb by using NM 101F and the human hinge BSpEI B primer. This fragment was cut with XhoI and BspEI enzymes and ligated in the XhoI/BspEI-digested plasmid containing VH of Ab 14C5 and CH1 region of PH1. A variant of the ch(Fab')_2_ was made with the His-tag on the light chain instead of on the heavy chain. This was done by exchanging the gene fragments 3^′^ of the BspEI between the heavy- and light-chain expression plasmids using a BspEI/PvuI digest.

### Production and derivatization

Light- and heavy-chain expression plasmids were transiently cotransfected in HEK293T cells by the Ca_3_(PO_4_)_2_ precipitation method [25,26]. Enzymatic digestion of chAb into chF(ab')_2_ and chFab was performed using the Pierce® F(ab')_2_ and Fab Preparation Kit (Thermo Scientific, Erembodegem, Belgium) according to the manufacturer's instructions.

### Protein purification

Purification of the chFab 14C5 and the chF(ab')_2_ fragment was performed by cation-exchange chromatography and Ni^2+^ affinity chromatography as described before [26]. The chFab and chF(ab')_2_ were concentrated and desalted using Centricon centrifugal filter devices (Millipore, Brussels, Belgium) with a cutoff of 30 and 50 kDa, respectively. Purification of chAb 14C5 was performed on a protein A column (GE Healthcare, Diegem, Belgium) using 20 mM sodium phosphate running buffer (pH 7) and 0.1 M glycine (pH 2.7, Sigma-Aldrich, Bornem, Belgium) elution buffer. Eluted fractions were neutralized immediately with 0.1 M Tris (pH 9, 100 μl/ml elution buffer). Further purification was performed by size exclusion chromatography on a Superdex-200 column (Amersham, Bioscience, Uppsala, Sweden). All purification steps were performed at 4°C on an Akta purifier system (Amersham Bioscience, Uppsala, Sweden).

### Analysis of protein fractions and stability determination

Primary antibodies used for detection of the Ab on Western blot and in enzyme-linked immunosorbent assay (ELISA) experiments were mouse anti-human kappa Ab or mouse anti-his Ab (Sigma-Aldrich, Bornem, Belgium). Alkaline phosphatase-conjugated anti-mouse IgG1 Ab (Becton Dickinson-Pharmingen Biosciences, Erembodegem, Belgium) was used as secondary Ab with the NBT/BCIP kit (Invitrogen, Life Technologies, Merelbeke, Belgium) for Western blot detection and *p*-nitrophenyl phosphate (Sigma-Aldrich, Bornem, Belgium) for the detection in the ELISA. Protein purity was analyzed by instant Coomassie brilliant blue dye (Expedeon, Cambridgeshire, UK), and specific detection of proteins was performed by immunodetection on a nitrocellulose membrane after Western blotting. Protein concentration was determined with the Micro BCA Protein Assay Kit (Pierce, Rockford, IL, USA) according to the manufacturer's instructions. All constructs were stored in different conditions (in phosphate-buffered saline (PBS), in cell medium at 37°C and 4°C, and in mouse serum at 37°C). Stability of the constructs was evaluated by SDS-PAGE followed by Coomassie staining and Western blot detection.

### Cellular ELISA

A549 and Colo16 cells (10^4^ cells/well) were coated in a 96-well plate and maintained at 37°C and 5% CO_2_ until confluence. Cells were gently washed with 300 μl PBS (Lonza, Verviers, Belgium). After fixation of cells with 100 μl PBS/acetone (2:3 (*v*/*v*), Sigma-Aldrich, Bornem, Belgium), cells were washed again. The wells were blocked with 2% PBS-M (2 g non-fat dry milk powder (Nestlé, Brussels, Belgium)/100 ml PBS) for 1.5 h. A ½ sample dilution series was prepared in PBS-M and added to the cells in triplicate. After 1.5 h of incubation at 37°C, the cells were washed three times and incubated with the primary detection Ab for 1 h at 37°C, washed again for three times, and incubated with the secondary detection Ab for 1 h at 37°C and washed again. Finally, the cells were washed a last time with substrate buffer (1 mM MgCl_2_ + 50 mM Tris (pH 9.5)), and detection was completed by adding detection buffer (1 tablet *p*-nitrophenyl phosphate (Sigma-Aldrich, Bornem, Belgium)/5 ml substrate buffer). Light absorbance at 405 nm was measured after approximately 2 h at 37°C in a microplate reader (Bio-Rad, Eke, Belgium).

### Radioactive labeling and radiochemical stability

14C5 chAb and its derivatives were labeled with ^125^I or ^131^I (PerkinElmer, Zaventem, Belgium) by incubating 10 min at room temperature using the Iodo-Gen method [27]. Separation of protein-bound iodine from free iodine was accomplished by purification over a PD10 column (GE Healthcare, Diegem, Belgium). Radiochemical stability of the iodinated molecules was assessed by instant thin layer chromatography (iTLC; SG strips, Pall Corporation, Zaventem, Belgium), by analysis of phosphorescence image after SDS-PAGE, and by size-exclusion high-performance liquid chromatography (HPLC) using a Shodex KW 802.5 with a KW-G guard column (Thomson Instrument Company, Oceanside, CA, USA). iTLC was performed using 20 mM citric acid (pH 5.0, Sigma-Aldrich, Bornem, Belgium) as eluent, strips were divided into six parts, and radioactivity was counted with a NaI(Tl) scintillation detector (PerkinElmer, Zaventem, Belgium). The labeled compounds were incubated in medium (4°C and 37°C), in PBS (4°C and 37°C), and in mouse serum (37°C) and were analyzed for radiochemical purity after several time periods.

### Saturation binding assay

A dilution series of the labeled Ab and fragments was added to 0.5 × 10^6^ cells and incubated for 2 h at 4°C. After centrifugation (15 s, 5,000×*g*) and washing with ice-cold PBS (three times), the cell pellet was counted for radioactivity using a gamma counter (Cobra II, PerkinElmer, Zaventem, Belgium). The dissociation constants (K_D_) were estimated (GraphPad Prism Software 5.0, San Diego, CA, USA) to determine the binding capacity of the Ab and fragments. The non-specific binding curve was obtained in two different ways: by incubation of the labeled molecule with the negative control cell line Colo16 and by incubation in the presence of unlabeled mAb 14C5 with the antigen-positive cell line A549.

### Biodistribution

Nu/Nu athymic mice of 5 to 9 weeks old (Charles River, L'Arbresle, France) were subcutaneously inoculated with 10^6^ tumor cells into the flank. When tumors reached a size of 0.5 to 1.0 g, biodistribution studies were performed. Mice were injected in a tail vein with 5 μg of 0.185 to 0.296 MBq ^131^I-labeled chAb or derivatives or negative control chAb MabThera (kind gift of Ghent University Hospital, Ghent, Belgium). Groups of four to five mice were sacrificed 0.02, 1, 3, 6, 8, 10, 24, 48, 72, and 168 h after injection. Tumor and organs were removed, washed in PBS, blotted dry, and weighed. Radioactivity in tumor, organs, and blood was detected with a NaI(Tl) scintillation detector (PerkinElmer, Zaventem, Belgium). Blood clearance studies were performed in 5- to 9-week-old Naval Medical Research Institute (NMRI) mice (*n* = 3) (Charles River, L'Arbresle, France). Data were analyzed by a two-phase exponential curve fit (GraphPad Prism 5.0). All animal experiments were approved by the Animal Experimental Ethical Committee of Ghent University Hospital (ECD 09/09). A non-parametric Mann–Whitney *U* test was used for statistical comparison.

## Results

### Construction and production of 14C5 chAb and derivatives

**Figure 1:**
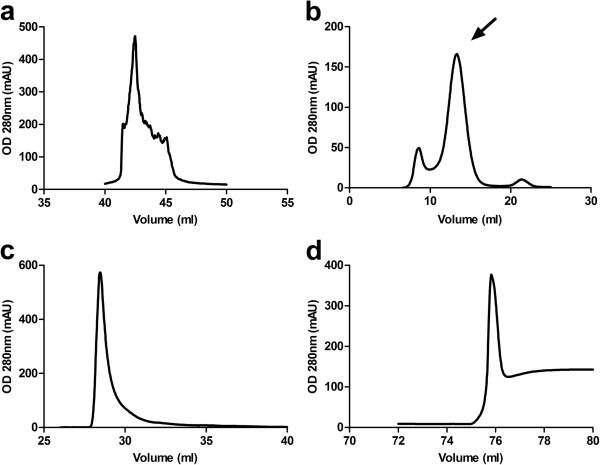
**Purification of chAb and chF(ab')**_**2 **_**14C5 produced by transfected HEKT293T cells.** Protein A elution of chAb 14C5 (**a**), gel filtration elution of chAb 14C5 (arrow indicates the chAb 14C5) (**b**), cation-exchange chromatography elution of chF(ab')_2_ (**c**) and Hi-Trap elution of chF(ab')_2_ (**d**). OD, optical density.

**Figure 2:**
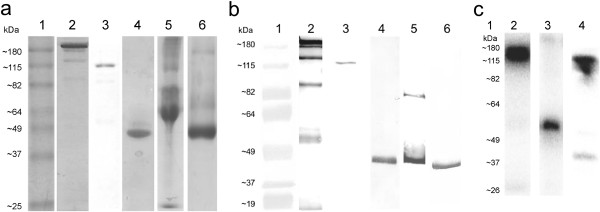
**SDS-PAGE analysis of the produced chimeric antibody and fragments 14C5.** Visualized using Coomassie brilliant blue dye (**a**) and specific visualization by Western blotting detected by mouse anti-human kappa antibody (lanes 2, 4, 5, and 6), mouse anti-His antibody (lane 3), followed by anti-mouse IgG1 alkaline phosphatase (**b**): lane 1, protein marker; lane 2, ch antibody; lane 3, enzymatically produced chF(ab')_2_; lane 4, enzymatically produced chFab; lane 5, recombinant chF(ab')_2_; lane 6, recombinant chFab (**a**, **b**). Phosphorescence image after SDS-PAGE: lane 1, estimated protein ladder based on non-radioactive protein marker; lane 2, ^125^I-ch antibody; lane 3, ^125^I-chFab; lane 4, ^125^I-chF(ab')_2_ 14C5 (**c**).

Chimeric mAb 14C5 derivatives were generated by recombinantly removing the 14C5 variable domains from the mouse antibody and fusing them to the constant domains of human IgG1. A full-size ch IgG1 was made by fusing the 14C5 V_H_ domain gene to the C_H_1 domain of a human IgG1 heavy-chain gene. Similarly, the 14C5 V_L_ domain gene was attached to the C_L_ of a human IgG1 light-chain gene. Both 14C5 gene constructs were integrated into the pES33 mammalian expression vector and were co-expressed in the mammalian HEK293T cell line. The cell supernatant containing the 14C5 chAb could be purified to a high degree by affinity purification on a protein A column, followed by polishing on a size-exclusion column (Figure 1a,b). Western blot analysis and Coomassie staining demonstrated that the 14C5 chAb was correctly produced and remained stable (Figure 2a,b, lane 2). Chimeric F(ab')_2_ and Fab fragments could then be successfully generated by pepsin and papain enzymatic digestion, respectively (Figure 2a,b, lanes 3 and 4). In order to simplify the production of these derivatives, a recombinant construct for 14C5 ch (Fab')_2_ was generated by removing the CH2 and CH3 domain genes from the heavy-chain construct. Also, removing the hinge-region gene from the heavy-chain vector led to a construct for a chFab 14C5. To facilitate purification, a C-terminal E-tag or His_6_-tag was added to the genes. After co-expressing the respective heavy- and light-chain vectors in HEK293T cells, the chAb derivatives were purified by cation-exchange chromatography followed by His-tag affinity purification (Figure 1c,d). However, Coomassie staining and Western blot analysis revealed that the majority of the chF(ab')_2_ was present in the form of dissociated F(ab') fragments (Figure 2a,b, lane 5). In an attempt to aid the formation of disulfide bridges, the F(ab') fragments were incubated with increasing concentrations of oxidized glutathione. However, this did not result in the formation of functional F(ab')_2_ dimers. Additionally, removing the His_6_-tag from the heavy chain did not result in improved dimerization of F(ab') fragments. In contrast, the proteolytically obtained chF(ab')_2_ (approximately 110 kDa, Figure 2a,b, lane 3) and the enzymatically and recombinantly produced chFab 14C5 fragment (approximately 50 kDa, Figure 2a,b, lanes 4 and 6) remained stable. Further *in vitro* and *in vivo* experiments with 14C5 chAb derivatives were performed with recombinantly produced chFab and proteolytically obtained chF(ab')_2_.

### Cellular ELISA

**Figure 3:**
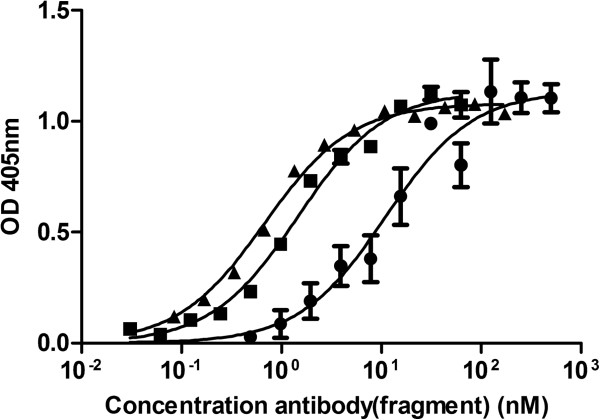
**Saturation binding experiment of the chF(ab**'**)**_**2 **_**(triangle), ch antibody (square), and chFab (circle) 14C5.** By cellular ELISA on A549 cells with mouse anti-human kappa antibody (IgG1) and anti-mouse IgG1-alkaline phosphatase. Data are expressed as mean ± SD (*n =* 3)*.*

The affinity of the chAb derivatives was determined through cellular ELISA on the α_v_β_5_-expressing A549 cell line. The α_v_β_5_^−^ Colo16 cell line served as a negative control. The *K*_D_ for the 14C5 chAb and its fragments were determined from the fitted specific binding curves (Figure 3). Due to their bivalent nature, 14C5 chF(ab')_2_ and chAb (*K*_D_ = 0.6 ± 0.05 nM and *K*_D_ = 1.4 ± 0.11 nM, respectively) showed a tenfold increase in relative affinity in comparison to the monovalent chFab 14C5 (*K*_D_ = 11 ± 2.34 nM).

### Radioactive labeling and radiochemical stability

Radioiodination of the chAb and fragments resulted in a typical yield between 75% and 85%. All labeled compounds showed a radiochemical purity of more than 95% as verified by iTLC and HPLC. No free iodine was present after HPLC and phosphorescence imaging after SDS-PAGE. No aggregation or degradation of ^125^I-labeled chAb 14C5 was observed (approximately 150 kDa, Figure 2c, lane 2). The ^125^I-chFab (approximately 50 kDa, Figure 2c, lane 3) and ^125^I-chF(ab')_2_ (approximately 110 kDa, Figure 2c, lane 4) revealed a low incidence of aggregation (>180 kDa) and degradation (approximately 37 kDa), accounting for less than 5% of the total sample radioactivity. All labeled constructs remained stable in several conditions (in PBS, medium, and mouse serum, at 4°C and 37°C) over a longer period with a radiochemical purity of more than 78% over a period of 3 days.

### Saturation binding assay

**Figure 4:**
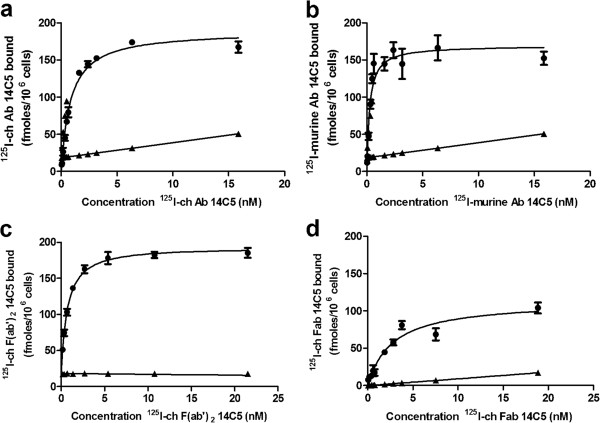
**Saturation binding assay of iodinated derivatives of the mAb 14C5 to A549 tumor cells*.*** Specific (circle) and non-specific binding (triangle) (binding to negative control Colo16 cell line) of ^125^I-chAb 14C5 (**a**), of ^125^I murine Ab 14C5 (**b**), of ^125^I-chF(ab')_2_ 14C5 (**c**), and of ^125^I-chFab 14C5 (**d**). Data are expressed as mean ± SD (*n* = 3)*.*

The affinity of the three ^125^I-radiolabeled 14C5 chAb derivatives was compared in a saturation binding assay on A549 cells (Figure 4). The *K*_D_ was determined at 1.19 ± 0.19 nM for the ^125^I-labeled chAb, 0.27 ± 0.05 nM for the parent murine Ab, 0.68 ± 0.10 nM for the chF(ab')_2_, and 2.11 ± 0.58 nM for the chFab. The non-specific binding curve obtained with the negative control Colo16 cells was similar to the non-specific curve obtained with unlabeled mAb 14C5 incubated on α_v_β_5_^+^ A549 cells.

### Biodistribution

**Figure 5:**
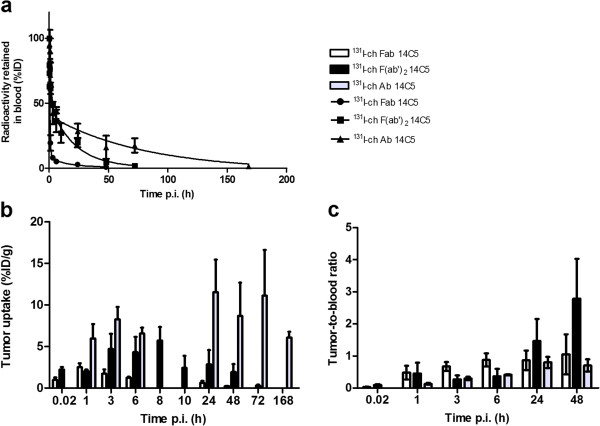
**Blood clearance in NMRI mice and tumor uptake and tumor-to-blood ratio in A549 lung tumor-bearing mice.** Blood clearance of ^131^I-labeled chFab, chF(ab')_2_, and chAb 14C5 in NMRI mice (**a**). Tumor uptake (%ID/g) (**b**) and tumor-to-blood ratio (**c**) of ^131^I-labeled chAb, chF(ab')_2_, and chFab 14C5 in A549 lung tumor-bearing mice. Values are corrected for decay of the radionuclide. Data are expressed as mean ± SD (*n* = 3 to 5)*.*

**Figure 6:**
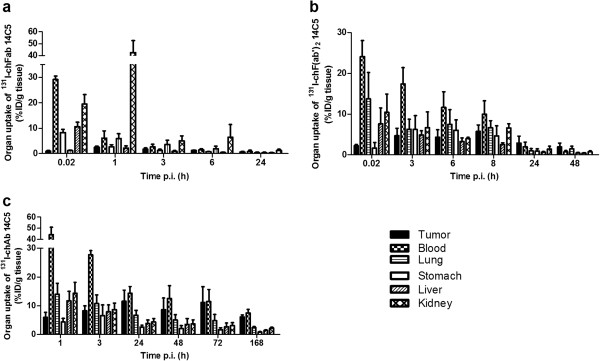
**Organ uptake of **^**131**^**I-chFab, **^**131**^**I-chF(ab')**_**2**_**, and **^**131**^**I-chAb 14C5.** Organ uptake (%ID/g tissue) of ^131^I-chFab (**a**), ^131^I-chF(ab')_2_ (**b**), and ^131^I-chAb 14C5 (**c**) in A549 lung tumor-bearing mice. Values are corrected for decay of the radionuclide. Data are expressed as mean ± SD (*n* = 3 to 5).

**Figure 7:**
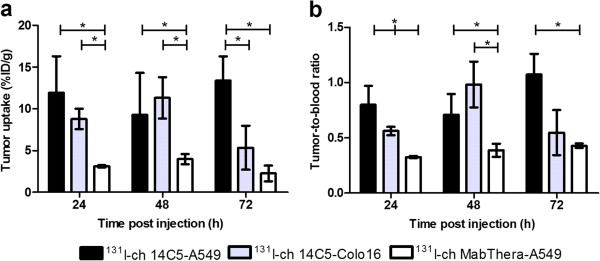
**Tumor uptake and tumor-to-blood ratio of **^**131**^**I-labeled chAb 14C5 and **^**131**^**I-labeled ch MabThera.** Tumor uptake (%ID/g) (**a**) and tumor-to-blood ratio (**b**) of ^131^I-labeled chAb 14C5 in A549 lung tumor-bearing mice and in Colo16-bearing mice and ^131^I-labeled ch MabThera in A549 lung tumor-bearing mice. Values are corrected for decay of the radionuclide. Data are expressed as mean ± SD (*n* = 3 to 5). The asterisk indicates *p* < 0.05, non-parametric Mann–Whitney *U* test.

In order to determine the pharmacokinetic properties of the 14C5 chAb and its derivatives, blood clearance and biodistribution studies were performed. The blood clearance for all molecules was determined after injection of 5 to 10 μg of ^131^I-labeled product in NMRI mice (Figure 5a). The estimated *t*_1/2_α was 19.6, 42.2, and 68.1 min for ^131^I-labeled chFab, chF(ab')_2_, and chAb, respectively. Furthermore, the *t*_1/2_β values for chFab, chF(ab')_2_, and chAb were estimated at 575.9, 867.8, and 3305 min, respectively. Subsequently, A549 tumor-bearing athymic mice were injected with the ^131^I-labeled chAb and its derivatives to determine the biodistribution (see Additional file 2). Maximum tumor uptake was reached at 1 h post injection (p.i.) (2.51 ± 0.46%ID/g) for ^131^I-chFab 14C5, at 8 h p.i. (5.73 ± 1.62%ID/g) for ^131^I-chF(ab')_2_, and at 24 h (11.56 ± 3.88%ID/g) for ^131^I-chAb (Figures 5b and 6). Total radiation towards the tumor was assessed by integration of tumor uptake over time. This resulted in area under the curve (AUC)_[0 → 2d]_ of 1.57%ID∙ d∙ g^−1^, AUC_[0 → 3d]_ of 6.74%ID∙ d∙ g^−1^, and AUC_[0 → 7d]_ 62.75%ID∙ d∙ g^−1^ for ^131^I-chFab, ^131^I-chF(ab')_2_, and ^131^I-chAb 14C5, respectively. Taking the amount of labeled compound in blood into account, the tumor-to-blood ratios (T/Bs) were calculated, which is an important characteristic for radioimmunodiagnosis (RID). The highest T/B of ^131^I-chFab was 0.88 from 3 h till 24 h p.i. The ^131^I-chF(ab')_2_ showed the highest T/B after 24 h till 72 h p.i. with a maximum of 2.79 ± 1.24 at 48 h p.i. The T/B of ^131^I-chAb ranged from 0.70 to 0.94 between 24 and 168 h p.i. (Figures 5c and 6). Another crucial property in RID and radioimmunotherapy (RIT) is the specificity of the tracer and the expression of the antigen. Exposure of normal tissue to radiation needs to be minimal to avoid non-specific damage. The highest non-specific uptake in the kidneys was seen with the ^131^I-chFab fragment 1 h p.i. The non-specific uptake of the tracer after 24 h p.i. in the lung, stomach, spleen, liver, and kidneys were markedly more elevated for the chAb 14C5 compared to its derivatives (Figure 6). Biodistribution of ^131^I-14C5 chAb in α_v_β_5_^−^ Colo16 tumor-bearing mice revealed a high initial uptake at 24 to 48 h p.i., which decreased significantly at 72 h p.i. (*p* < 0.05, non-parametric Mann–Whitney *U* test; Figure 7a). This is in contrast to the uptake seen in A549 tumors which was highest at 24 h p.i., remained high until 72 h p.i., and decreased only at 168 h p.i. (Figure 7a). The T/Bs in the Colo16 model also illustrate the diminished tumor retention as compared to the specific A549 tumor model (Figure 7b). The maximum Colo16 tumor uptake was 11.32 ± 2.48%ID/g at 48 h p.i., and the T/Bs increased to 1 (Figure 7). Additionally, experiments with a labeled negative control chAb, ^131^I-MabThera, demonstrated a significant lower uptake in the positive A549 tumor (maximum tumor uptake <5%ID/g) in comparison to the specific chAb 14C5 uptake A549 tumors (*p* < 0.05, non-parametric Mann–Whitney *U* test; Figure 7).

## Discussion

Chimeric Ab derivatives of the anti-α_v_β_5_ mAb 14C5 were recombinantly produced to reduce the risk of generating a HAMA response in humans. Chimerization is the first major and the most reliable step towards a more humanized therapeutic Ab molecule [28]. Complementarity-determining region grafting could decrease immunogenicity further but often results in reduced affinity or specificity for the target antigen. Safeguarding the desired characteristics involves several labor-intensive trial-and-error approaches [29,30]. Integrin α_v_β_5_ expression is involved in several crucial steps of cancer progression such as cell adhesion, angiogenesis, and metastasis [31,32]. Overexpression of α_v_β_5_ has been demonstrated not only on several tumor types, but also on non-transformed tumor-promoting cells from the tumor stroma, such as cancer-associated fibroblasts [33] and cells from the neovasculature [11,14,34]. These accessory cells are less likely to lose antigen expression, making α_v_β_5_ a very attractive target for Ab-based RID and/or RIT. The murine mAb 14C5 was shown to hold great potential for these applications, given the tenfold higher affinity for α_v_β_5_ of the 14C5 mAb (*K*_D_ = 0.19 ± 0.07 nM) compared to the commercially available murine P1F6 mAb [13,14,17,35]. However, despite its promising characteristics, the use of mAb 14C5 in humans might provoke a HAMA response due to its murine origin. HAMA responses generally lead to a reduced therapeutic effectiveness and can lead to potentially life-threatening severe allergic reactions. Replacing mouse Ab constant domains with their human counterparts in chimeric constructs generates less anti-chimeric Abs at lower titers as compared to HAMA in patients given the parent murine Ab [36]. A chimeric human IgG1 mAb 14C5 was recombinantly constructed, produced, and purified. After confirmation of its stability, the affinity of recombinantly produced chAb 14C5 for the integrin α_v_β_5_ was determined by cellular ELISA assays and by saturation binding assays with radioiodinated chAb. Both experiments revealed a similar and high affinity of the chAb for its antigen (*K*_D_ = 1.4 ± 0.11 nM and *K*_D_ = 1.19 ± 0.19 nM, respectively). Although the dissociation constant for the murine 14C5 mAb was ten times lower [13] compared to the chAb, this still puts the chAb 14C5 on par with the P1F6 mAb. Blood clearance studies of ^131^I-chAb 14C5 conducted in NMRI mice revealed negligible differences in *t*_1/2_α and *t*_1/2_β in comparison with the murine mAb 14C5 (*t*_1/2_α = 118 min and *t*_1/2_β = 4067 min) [13], since human as well as mouse IgG binds the mouse neonatal Fc receptor (FcRn) which regulates serum half-lives of IgG in both species. However, significantly different clearance values are expected in human patients, since human FcRn binds to human but not to mouse IgG, resulting in longer retention of the chAb in the blood [37]. The highest tumor uptake for chAb as well as for the murine mAb 14C5 [13,17] was seen at 24 h p.i. of approximately 10%ID/g, which corresponds to other high-affinity Abs, such as the humanized anti-α_v_β_3_ mAb Abegrin™ (14%ID/g at 24 h p.i.) [38]. In mice bearing the α_v_β_5_^−^ Colo16 squamous tumor, an initial high uptake of chAb 14C5 (approximately 10%ID/g at 24 and 48 h p.i.) was observed which decreased more rapidly compared to the uptake in the α_v_β_5_^+^ A549 tumor. In contrast, a negative control chAb MabThera showed significant less uptake (<5%ID/g, *p* < 0.05) in the α_v_β_5_^+^ A549 tumor in comparison with the uptake of chAb 14C5 in both α_v_β_5_^+^ and α_v_β_5_^−^ tumor types. Additionally, staining of different human tumor tissues with mAb 14C5 demonstrated tumor cell membrane and/or tumor surrounding stroma staining [14]. Tumor sections without tumor cell membrane staining did reveal stroma staining which was co-localized with a fibroblast-specific marker (data not shown). Consequently, expression of integrin α_v_β_5_ on cancer-associated fibroblasts (CAFs) was confirmed. Therefore, the uptake of chAb 14C5 seen in α_v_β_5_^−^ Colo16-bearing animals is attributed to binding on CAFs in the tumor stroma and/or on neo-vasculature of the tumor. These results confirm α_v_β_5_ as a promising target for RID and RIT. For RID smaller fragments with good tissue penetration and a fast clearance are needed, while RIT requires a good therapeutic index without too much collateral damage. 14C5 chAb Fab and F(ab')_2_ fragments were generated in order to examine their pharmacokinetic properties and find the best format for RID/RIT applications. Both the chFab and chF(ab')_2_ obtained from proteolytic cleavage of the chAb 14C5 as well as the recombinantly produced chFab were correctly produced and remained stable. However, expression of the chF(ab')_2_ vectors did not result in significant amounts of correctly dimerized chF(ab')_2_. Analysis on SDS-PAGE suggests that the stabilizing disulfide bridges between chFab' fragments were not properly formed. Procedures to oxidize the cysteine SH groups in order to form chF(ab')_2_ or the removal of peptide tags on the heavy chain did not result in increased yields of stable recombinant chF(ab')_2_, leaving only the proteolytically obtained chF(ab')_2_ for further experiments. In cellular ELISA, the avidity of the bivalent 14C5 chF(ab')_2_ (*K*_D_ = 0.6 ± 0.05 nM) for the α_v_β_5_^+^ A549 lung tumor cell line was ten times higher compared to the monovalent 14C5 chFab (*K*_D_ = 11 ± 2.34 nM). However, saturation binding experiments with ^125^I-labeled compounds revealed a less pronounced difference between the affinity of both fragments, with a *K*_D_ = 0.68 ± 0.10 nM and *K*_D_ = 2.11 ± 0.58 nM for the ^125^I-chF(ab')_2_ and chFab 14C5, respectively. Similar results were obtained for the 14C5 murine F(ab')_2_ (*K*_D_ = 0.37 ± 0.10 nM) and Fab (*K*_D_ = 2.25 ± 0.44 nM) [17], indicating that the chimerization of the fragments had no influence on the *in vitro* binding characteristics. *In vivo*, for the chFab 14C5 fragment, the peak tumor-to-blood ratio (0.9) was reached at 6 h p.i. After 24 h the Fab was largely cleared from the system, which makes it an interesting probe for RID, despite high non-specific kidney uptake caused by renal clearance of the small fragment (<60 kDa). Behr et al. [39] demonstrated that administration of cationic amino acids, such as d-lysine, reduced the renal uptake of small peptides. The ^131^I-chF(ab')_2_ demonstrated improved tumor-to-blood ratios compared to the Fab fragment, although the molecule remained in circulation longer. The ^131^I-chF(ab')_2_ did however clear faster than the intact chAb. The AUC value is an indication for the total amount of radiation in the tumor. The chAb 14C5 AUC value (AUC_[0 → 7d]_ 62.75%ID∙ d∙ g^−1^) was higher than the AUC value of its fragments, suggesting a higher therapeutic efficacy. However, the long residence time of the intact Ab in the blood may augment the risk of non-specific radiation damage (e.g., bone marrow). Consequently, the faster clearing chF(ab')_2_ might be an attractive alternative if dose-limiting toxicity occurs after administration of the intact Ab. The AUC values may be further improved by using radiometals such as ^111^In and ^90^Y, which have been shown to improve tumor retention times and AUC values of radiolabeled immunologicals [40]. Internalization studies with ^111^In-14C5 mAb have already demonstrated extended tumor retention times compared to radioiodinated Ab 14C5 [35].

## Conclusions

In conclusion, this study demonstrated that chimerization of mAb 14C5 had little effect on the properties of the mAb. The characteristics of the 14C5 chAb are promising for RIT and monitoring, while the chF(ab')_2_ and chFab 14C5 have a high potential for RID of cancer. In addition to these fragments, other Ab constructs could be created with improved characteristics for both RID and RIT. Smaller constructs, such as diabodies and nanobodies for example, provide high penetration into the tumor and a rapid blood clearance, which is highly advantageous for RID. Larger constructs, such as minibodies for example, provide longer target retention and could be used in RIT [41].

## Competing interests

The authors declare that they have no competing interests.

## Authors’ contributions

CD designed and carried out the experimental studies and wrote the manuscript. SS contributed to the conception and design of the study and critically contributed to and revised the manuscript. LM and SN contributed to the carrying out of experimental studies and to the revision of the manuscript. JH participated in the design of protein purification. FDV participated in the design of the study and helped to draft the manuscript. All authors read and approved the final manuscript.

## Supplementary Material

Additional file 1 **Primers used in the construction of chimeric derivatives of antibody 14C5 expression plasmids.** DNA sequences of the primers (5^′^ to 3^′^). Click here for file

Additional file 2 **Biodistribution.** Biodistribution of ^131^I-labeled chimeric antibody 14C5 and fragments (%ID/g tissue) in athymic mice bearing an A549 tumor. Click here for file
