# Supplementary material for: Generation and in vivo characterization of a chimeric αvβ5-targeting antibody 14C5 and its derivatives
Source: EJNMMI Res. 2013 Apr 4;3:25. doi: 10.1186/2191-219X-3-25 (PMC3626673; doi:10.1186/2191-219X-3-25)
Supplement: Additional file 1 — Primers used in the construction of chimeric derivatives of antibody 14C5 expression plasmids. DNA sequences of the primers (5′ to 3′). [file 2191-219X-3-25-S1.pdf]

Additional file 1. Primers used in the construction of chimeric derivatives of antibody 14C5 expression plasmids

Name	Sequence 5’ to 3’
**NM 101 F**	CAACGTGCTGGTTATTGTGCTGTC
**14C5 VH B SOE**	AGACCGATGGACCCTTGGTGGAGGCTGAGGAGACGGTGACTGAGG
**PH1 CH1 F SOE**	AGGAACCTCAGTCACCGTCTCCTCAGCCTCCACCAAGGGTCCATC
**NM 263 B**	TATGGATCCTTATCCGGAGGGCCCTGCGGCCGCACAAGATTTGGGCTC
**14C5 VL B SOE**	AGACAGATGGTGCAGCCACAGTTCGTTTGATTTCCAGCTTGGTGCCTCCAC
**PH1 CL F SOE**	TGGAGGCACCAAGCTGGAAATCAAACGAACTGTGGCTGCACCATCTGTC
**NM 264 B**	TATGGATCCTTATCCGGAGGGCCCACACTCTCCCCTGTTGAAGCTC
**CH1 Hi B**	GCACGGTGGGCATGTGTGAGTTTTGTCACAAGATTTGGGCTCAACTTTC
**CH2 F**	CAAAACTCACACATGCCCACCGTGCCCAGCACCTGAACTCCTGGGGGGAC
**CH2 B**	TGTGGTTCTCGGGGCTGCCCTTTGGCTTTGGAGATGGTTTTC
**CH3 F**	AAACCATCTCCAAAGCCAAAGGGCAGCCCCGAGAACCACAGG
**CH3 B**	ATGGATCCTTATTTACCCGGAGACAGGGAGAGGC
**Hum hinge BspEI B**	TCCGGATGGGCACGGTGGGCATGTGTGAGTTTTGTC
