# Supplementary material for: Generation and in vivo characterization of a chimeric αvβ5-targeting antibody 14C5 and its derivatives
Source: EJNMMI Res. 2013 Apr 4;3:25. doi: 10.1186/2191-219X-3-25 (PMC3626673; doi:10.1186/2191-219X-3-25)
Supplement: Additional file 2 — Biodistribution. Biodistribution of 131I-labeled chimeric antibody 14C5 and fragments (%ID/g tissue) in athymic mice bearing an A549 tumor. [file 2191-219X-3-25-S2.pdf]

Additional file 2. Biodistribution of ^{131}I-labeled chimeric antibody 14C5 and fragments (%ID/g tissue) in athymic mice bearing an A549 tumor**.** Data are expressed as mean ± S.D. *(n=4-5), (nd; not determined)*

Ab derivative	Tissue and tumor-to-blood ratio	Time post injection (h)									
		0.02	1	3	6	8	10	24	48	72	168
ChFab	Tumor	0.99±0.29	2.51±0.46	1.73±0.51	1.26±0.10	nd	nd	0.63±0.25	0.23±0.03	nd	nd
	Blood	29.15±1.38	6.14±2.83	2.70±1.06	1.50±0.45			0.84±0.50	0.28±0.15		
	Lung	8.31±1.20	2.61±0.93	1.27±0.45	0.72±0.13			0.39±0.28	0.15±0.06		
	Stomach	1.20±0.19	5.92±1.94	3.56±1.72	1.90±1.10			0.32±0.26	0.08±0.03		
	Spleen	4.41±1.09	2.12±0.86	0.90±0.34	0.51±0.25			0.31±0.21	0.09±0.07		
	Liver	10.64±1.75	2.22±0.82	0.90±0.32	0.49±0.15			0.34±0.16	0.16±0.11		
	Kidney	19.50±3.70	42.54±10.09	5.03±1.97	6.48±5.00			1.25±0.47	0.64±0.28		
	Small intestine	0.85±0.09	1.42±0.26	0.71±0.17	0.36±0.02			0.08±0.05	0.03±0.01		
	Large intestine	0.37±0.09	0.60±0.18	0.67±0.16	0.40±0.08			0.08±0.05	0.04±0.02		
	Tumor-to-blood	0.03±0.01	0.48±0.21	0.67±0.14	0.88±0.21			0.87±0.30	1.05±0.63		
ChF(ab’)$_2$	Tumor	2.20±0.32	2.05±0.16	4.72±1.81	4.30±1.86	5.73±1.62	2.43±1.44	2.86±1.72	1.94±0.95	0.28±0.13	nd
	Blood	24.08±3.97	19.74±4.99	17.35±4.08	11.67±3.84	9.97±3.29	6.74±1.72	1.95±1.18	0.70±0.34	0.17±0.14	
	Lung	13.71±6.51	7.18±1.62	6.28±2.45	7.44±3.65	6.65±1.72	13.16±0.10	0.95±0.55	1.43±0.70	0.09±0.03	
	Stomach	1.64±1.36	6.29±2.58	6.24±3.41	6.00±2.55	4.63±2.49	5.37±2.97	0.88±0.56	0.38±0.20	0.14±0.11	
	Spleen	2.66±1.44	2.88±0.96	2.62±1.88	3.00±1.37	2.16±0.36	2.06±0.01	0.71±0.44	0.32±0.14	0.13±0.02	
	Liver	7.59±3.93	4.82±0.74	4.87±1.04	3.25±0.90	2.51±0.44	2.33±0.01	0.65±0.13	0.38±0.11	0.16±0.03	
	Kidney	10.44±4.44	14.19±3.27	6.62±3.92	3.96±0.36	6.59±0.99	5.13±0.87	1.38±0.76	0.69±0.27	0.30±0.17	
	Small intestine	1.18±1.12	3.97±3.03	5.03±2.92	1.82±0.17	1.43±0.64	1.25±0.36	0.28±0.07	0.17±0.08	0.07±0.01	
	Large intestine	0.49±0.30	0.51±0.31	1.88±0.30	1.52±0.48	1.47±0.71	1.34±0.51	0.32±0.06	0.22±0.11	0.06±0.03	
	Tumor-to-blood	0.09±0.03	0.46±0.33	0.27±0.12	0.37±0.23	0.58±0.03	0.34±0.12	1.47±0.69	2.79±1.24	1.99±0.99	
ChAb	Tumor	nd	5.97±1.74	8.24±1.70	6.58±0.67	nd	nd	11.56±3.88	8.69±4.01	11.12±5.51	6.07±0.71
	Blood		44.27±6.53	27.74±1.52	15.79±1.08			14.33±2.34	12.49±4.48	11.50±4.19	7.46±1.29
	Lung		13.99±3.84	10.75±3.00	5.86±1.58			6.73±1.66	5.00±1.89	4.79±2.22	2.34±0.39
	Stomach		4.30±1.33	6.58±3.68	2.73±0.74			2.60±0.66	2.05±1.03	1.66±0.69	0.76±0.27
	Spleen		12.87±2.80	7.82±2.04	5.09±0.72			7.37±2.40	4.10±1.92	2.98±1.26	1.63±0.42
	Liver		11.74±3.28	7.88±2.45	4.99±1.97			3.84±1.15	3.44±2.12	2.67±1.35	1.44±0.17
	Kidney		14.36±3.84	8.64±2.26	4.85±0.79			4.36±1.21	3.65±1.46	3.05±1.01	2.15±0.30
	Small intestine		3.26±1.03	4.13±0.84	1.46±0.38			1.22±0.53	0.94±0.53	0.76±0.29	0.51±0.05
	Large intestine		1.18±0.11	2.04±0.41	1.61±0.40			0.73±0.27	0.69±0.59	0.49±0.13	0.29±0.04
	Tumor-to-blood		0.13±0.03	0.30±0.05	0.42±0.02			0.80±0.17	0.71±0.19	0.94±0.35	0.83±0.13
